# Implementing a stepped-care approach in primary care: results of a qualitative study

**DOI:** 10.1186/1748-5908-7-8

**Published:** 2012-01-31

**Authors:** Gerdien Franx, Matthijs Oud, Jacomine de Lange, Michel Wensing, Richard Grol

**Affiliations:** 1 Trimbos-institute, Netherlands Institute of Mental Health and Addiction, PO Box 725, 3500 AS Utrecht, the Netherlands; 2 Scientific Institute for Quality of Healthcare, Radboud University Nijmegen Medical Centre, PO Box 9101, 114, 6500 HB Nijmegen, the Netherlands

## Abstract

**Background:**

Since 2004, 'stepped-care models' have been adopted in several international evidence-based clinical guidelines to guide clinicians in the organisation of depression care. To enhance the adoption of this new treatment approach, a Quality Improvement Collaborative (QIC) was initiated in the Netherlands.

**Methods:**

Alongside the QIC, an intervention study using a controlled before-and-after design was performed. Part of the study was a process evaluation, utilizing semi-structured group interviews, to provide insight into the perceptions of the participating clinicians on the implementation of stepped care for depression into their daily routines. Participants were primary care clinicians, specialist clinicians, and other healthcare staff from eight regions in the Netherlands. Analysis was supported by the Normalisation Process Theory (NPT).

**Results:**

The introduction of a stepped-care model for depression to primary care teams within the context of a depression QIC was generally well received by participating clinicians. All three elements of the proposed stepped-care model (patient differentiation, stepped-care treatment, and outcome monitoring), were translated and introduced locally. Clinicians reported changes in terms of learning how to differentiate between patient groups and different levels of care, changing antidepressant prescribing routines as a consequence of having a broader treatment package to offer to their patients, and better working relationships with patients and colleagues. A complex range of factors influenced the implementation process. Facilitating factors were the stepped-care model itself, the structured team meetings (part of the QIC method), and the positive reaction from patients to stepped care. The differing views of depression and depression care within multidisciplinary health teams, lack of resources, and poor information systems hindered the rapid introduction of the stepped-care model. The NPT constructs 'coherence' and 'cognitive participation' appeared to be crucial drivers in the initial stage of the process.

**Conclusions:**

Stepped care for depression is received positively in primary care. While it is difficult for the implementation of a full stepped-care approach to occur within a short time frame, clinicians can make progress towards achieving a stepped-care approach, particularly within the context of a QIC. Creating a shared understanding within multidisciplinary teams of what constitutes depression, reaching a consensus about the content of depression care, and the division of tasks are important when addressing the implementation process.

## Background

Since 2004, 'stepped-care models' have been adopted in several international evidence-based clinical guidelines on depression globally [1-3]. More recently, stepped-care approaches for depression have been found to be feasible in primary care for diverse patient populations. Stepped-care approaches can both generate well-being and reduce healthcare costs [4-10].

The key idea underpinning stepped depression care is that patients with sub-threshold and mild depression are offered interventions of low intensity, such as psycho-education, self help, counseling, physical exercise, or problem-solving treatment. Watchful waiting is also valid in this phase. For a patient who does not successfully respond to these approaches, or for patients whose symptoms are more severe, more intensive treatment options are appropriate. Antidepressants, psychotherapy, or electroconvulsion therapy (ECT), combined with case management and self-management strategies are preferred options for severe and chronic cases. One key aspect of the stepped-care approach is the monitoring of patient progress in order to recognize when it is necessary to step up to a more intensive treatment [5,6,11]. Despite the positive recommendations in guidelines, the embedding of stepped depression care in normal daily primary care asks for a paradigm shift that has not been fully achieved. This is illustrated by previous research, which found that antidepressant prescription rates remained high and unrelated to symptom severity, and that cost-effective alternatives for patients with mild depression are still underused [12-14].

Historically, the Netherlands has had a strongly developed primary care system, consisting of general medicine provided by primary care physicians (PCPs), paramedical and pharmaceutical care, nursing and supportive care, as well as non-specialised mental and social healthcare. Preventive and health educational activities are linked to these forms of care. The PCP is the central provider for all medical care, including mental healthcare, and the gatekeeper to specialist care. Each full-time PCP has a caseload of around 2,400 patients and is paid on a fixed annual fee basis per patient subscribed to the practice. Over the last decade, different measures have been launched to strengthen primary mental healthcare, such as increasing the numbers of, and capacity of social workers, and the introduction of specialised mental health nurses in about 25% of the general practices [15,16]. Yearly, between 11% to 13% of the adult population is registered by the PCP with a psychological problem or diagnosis [16]. Of those presenting to the PCP with a psychological problem, 3% to 4% present with depression. This figure has remained stable in the Netherlands between 2002 and 2008. The majority of patients with depression are treated by the PCP; less than 8% of cases are referred to a social worker, mental health nurse, primary care psychologist, or to a specialist. PCPs treat their patients mostly during a number of brief consultations of less than twenty minutes, and a large proportion of patients (up to 70% in 2008) are treated with antidepressant medication [16].

It has been recognized, that successful implementation of complex treatment approaches in healthcare, such as stepped care for depression, depends on a complex interplay of factors and overcoming several barriers to implementation. There have been a wealth of theories and models developed to explain the factors affecting implementation of innovations. The explanatory models can be categorized in the following manner: theories focusing on characteristics of individual professionals, theories on social influence or interpersonal factors, and theories on system characteristics such as organizational and economic factors [17,18]. Barriers and facilitators for change can be identified on six levels: the innovation itself, the individual professional, the patient, the social context, the organisational context, and the economic and political context [18]. A recently developed theory on implementation of innovations is the Normalization Process Theory (NPT), developed by May and Finch (2009), which offers a conceptual map for the evaluation of complex interventions [19]. According to NPT, there are four mechanisms that drive change: coherence, cognitive participation, collective action, and reflexive monitoring [19]http://normalizationprocess.co.uk/whatfor.aspx. The care system will only function seamlessly if all four constructs operate concurrently and are attended to [20]. More recently, Gunn *et al. *adapted the NPT theory for use in depression care [20].

In this study we present the findings of a qualitative process evaluation, within a controlled study looking at the effectiveness of a depression Quality Improvement Collaborative (QIC). The findings of the controlled intervention study are presented in another paper submitted for publication. Previously published uncontrolled observational data of the QIC demonstrated an improvement of stepped-care treatment within the teams participating in the QIC [21]. The qualitative process analysis presented here aims to add to the quantitative findings as it documents the way in which the intervention was received and implemented by clinicians, and identifies the factors associated with reception and implementation. Furthermore, by relating the findings to the NPT constructs, we were able to provide another layer to the findings. The constructs provide us with sensitizing concepts that could lead to a better understanding of the findings of this process evaluation, as well as guide additional recommendations on how to conduct implementation projects in depression care.

## Methods

### Study design

Alongside the QIC, an intervention study using a controlled before-and-after design was performed. The overall study protocol comprised an effectiveness study, a process evaluation, and a cost-effectiveness evaluation. The intervention group consisted of PCPs participating in the QIC, the parallel control group, providing care as usual, consisted of a selection of PCPs from practices participating in the Netherlands Information Network of General Practice (LINH). This database holds longitudinal and nationally representative data on morbidity, prescribing and referrals of about 350.000 individuals. Data collection in both groups covered a three-year period: from the beginning of 2006 (the year prior to the QIC) until end of 2008 (the year after the QICs ending). The primary outcome of the study was a change of antidepressant prescription rates to patients with a new diagnosis of depression in both groups. The qualitative process evaluation was directed at generating insight into the perceptions of the participating clinicians in the intervention group on the implementation of stepped care for depression into their daily routines. Data collection was obtained via group interviews, which were held between December 2006 and March 2008.

Ethics approval for the entire study protocol was provided by the METIGG, a national ethics committee in mental healthcare in the Netherlands.

### Setting and participants

Participants for the study were selected from thirteen multidisciplinary primary care teams participating in the depression QIC. These thirteen QIC teams had been recruited throughout the country by a national QIC project team on the basis of the following criteria: the team had a multidisciplinary structure, there was sufficient motivation and time for all members to participate, and a local team coordinator was available. Although team members sometimes had worked together in another context, most of them had not worked together as a depression team prior to the QIC. At the start of the QIC, all teams were asked to participate in the intervention study and the process evaluation, alongside their implementation work. Five teams did not wish to spend extra time on research activities and declined. Eight teams consented, consisting of PCPs, primary care psychologists, social workers, mental health nurses, physiotherapists, consulting psychiatrists and psychotherapists, local managers, and team coordinators.

### Intervention

The intervention consisted of a QIC aimed at the implementation of a stepped-care approach for depression in a multidisciplinary, primary care setting. The QIC was designed as a 'Breakthrough' QIC [22,23]. Three stepped-care improvement principles, designed by the QIC's national expert team and derived from the national clinical guidelines, were intended to guide the implementation processes [20]--patient differentiation, stepped treatment, and monitoring of treatment outcomes (Table 1).

**Table 1:** Stepped-care principles of the Depression Quality Improvement Collaborative

1.	Patient differentiation. The general practitioner diagnoses the patient, using the International Classification of Primary Care (ICPC) diagnosis P03 or P76 (Lamberts & Wood, 1990). The clinician classifies the depression to be either severe or non-severe, according to the criteria of the stepped-care model.
2.	Stepped treatment. Non-severely depressed patients are offered an intervention of low intensity as a first line treatment, such as: watchful waiting, psycho-education, self-help, counseling, brief psychotherapy, physical exercise. After six to twelve weeks, when response is insufficient, clinicians step up to a next level of intensity, antidepressant medication or cognitive behavioral psychotherapy.

3.	Outcome monitoring. The Beck Depression Inventory (BDI), a 21-item self-report inventory, for measuring the severity of depression, is used to monitor symptom severity. A score of 0 to 9 indicates a normal mood, patients with higher scores are monitored every six weeks until the score has returned to normal. Stepping up to higher intensity level treatments is considered in case of insufficient response.

A local team coordinator supported the team with the aim of structuring the implementation process. Local team coordinators received brief training from the QIC national expert team about the use of Plan-Do-Study-Act (PDSA) cycles and about the monitoring of stepped care and depression indicators in a Microsoft Office Excel work sheet. Both elements, PDSA-cycles and monitoring, are crucial elements of QICs and help to move the implementation process forward [24]. To assist the clinicians in applying the stepped-care principles into daily clinical practice, the QIC national expert team offered four national conference days for learning, seven meetings for quality improvement project managers, regular telephone contact, as well as working visits to all sites. The clinicians independently set up bi-monthly local team meetings for discussions about the translation of the principles into their work settings, and to exchange experiences, progress, and steps for further improvement. In addition, all individual clinicians had access to workshop sessions and to online materials, such as a depression toolkit describing evidence-based interventions. Funding for these support activities primarily came from external bodies; however, the primary care teams also independently co-financed a small portion of the project [21].

### Data collection

Data collection consisted of eight semi-structured group interviews with duration of 60 to 75 minutes with all participating multidisciplinary improvement teams. The interviews took place during the last half of the 15 months of the QIC. Group interviews with the multidisciplinary teams were appropriate given that there is limited knowledge about applying stepped-care principles for depression from the professional perspective, particularly with healthcare professionals coming from different backgrounds. The interviews therefore were expected to provide additional exploratory data that can enrich quantitative findings. The interviews were conducted by the researchers (GF, MO), following a topic list with questions related to the stepped-care changes made in clinical practice, the mechanisms and factors that influenced the change processes, and the impact of the changes on the care delivered as perceived by the respondents. The researchers had no relationship with the respondents prior to the interviews, but were familiar with the QIC work from holding former positions in other projects. The interviews were audio taped with consent of the participants and transcribed verbatim.

### Analysis

The interview transcripts were analysed independently by two coders (combinations of GF, MO, and JdL). The perspective of JdL, a qualitative research expert and the national project manager of the depression QIC, ensured that the data were interpreted and understood from different perspectives. To order the data, thematic coding was used with the help of MaxQda 2007, qualitative analysis software http://www.maxqda.com/. Samples of the coded fragments were compared and settled by consensus. As a result, a coding tree was built around the following key themes; the experiences with the QIC method, the changes made in the primary care practices, the factors influencing the change processes and the results of the change processes in terms of outcomes for patients and efficiency of care. Within these themes, different levels of codes were constructed. For example, within the theme of influencing factors, the code 'culture' was assigned, referring to the views within the teams on depression care. Within this code, sub-codes were drawn from the material, such as 'pro-activity,' 'openness and trust,' and 'views on depression,' the latter referring to transcripts in which respondents talked about how their personal concept of depression played a role in introducing stepped care within the team. Finally, the material was ordered for reporting around the research questions about how the stepped-care principles were applied and experienced, and which factors influenced this process. Because our goal was to capture groups' experiences, the findings are reported as the teams' perspectives on each of the stepped-care principles. The viewpoints of specific professional groups were only described when relevant.

The interpretations were discussed within the project team. The preliminary results were discussed with the respondents, approximately one year after the QIC's termination (member check). A researcher (MO) interviewed a member of each of the improvement teams by telephone. During these telephone interviews, team representatives were asked whether they agreed with the results from the qualitative interviews, if the analysis had missing information that was important for enriching the data, and if the results were applicable for their team. The interviews confirmed the results.

After this analysis, we used Gunn's NPT framework on depression [20] to help understand and further interpret the qualitative findings. Because this framework is a 'conceptual framework for implementing best practice depression care that is informed by NPT' we considered the additional use of the framework of interest to generate a more in-depth understanding of the stepped-care implementation process [20]. Gunn's depression framework is built on the four NPT mechanisms that drive change: coherence, cognitive participation, collective action, reflexive monitoring [17]; http://normalizationprocess.org). The mechanism of coherence refers to the way in which depression care is conceptualized by healthcare professionals, and implies that all actors should have a shared understanding of what constitutes depression and depression work. This shared understanding is necessary for adoption of an effective stepped-care model for depression in routine care. Cognitive participation outlines how professionals engage in depression care, and implies an agreement that depression care is part of routine care and that there is a shared set of diagnostic and treatment techniques. The third mechanism, collective action, is about how depression care is organized and what factors constrain and structure the depression care activities. The fourth mechanism of reflexive monitoring is the agreement between the clinicians on how depression care is appraised and the understanding about why the depression care happened as it did [20]. In our study, the four NPT-based constructs served to reframe our findings, to describe additional relevant issues to stepped-care approaches for depression, and to further elaborate on these issues.

## Results

Eighty clinicians and support staff working in eight primary care teams, expressed an intention to implement stepped depression care, introduced to them during the QIC. The participants consisted of PCPs (n = 20), primary care psychologists (n = 9), psychiatrists and psychologists consulting in primary care (n = 6), social workers (n = 11), physiotherapists (n = 5), specialized mental health nurses (n = 7), pharmacists (n = 2), local project managers (n = 10) and local staff or managers (n = 9) (Table 2).

**Table 2:** Distribution of participants between the QIC teams

Team identity	PCP	SMHN	PCP	SW	Pht	Pth/psy	Pharm	Pm	Other
1	1	1	1	1				1	

2	2	1	2	2	1	1		1	2

3	4	1	1	1		1		1	

4	1	1	1	1		1		2	

5	2	1		2	1	1		2	3

6	5	1	2	2	1			1	

7	1	1	1			1		1	2

8	4		1	2	2	1	2	1	1

total	20	7	9	11	5	6	2	10	9

PCP: primary care psychologist

SMHN: specialised mental health nurse

SW: social worker

Pht: physiotherapist or psychomotor therapist

Pth/psy: psychotherapist, psychiatrist, specialised psychologist

Pharm: pharmacist

Pm: local project manager

Other: manager and supportive staff

### Patient differentiation

The first stepped-care principle concerned the differentiation between two categories of patients: patients with severe symptoms and patients without severe symptoms. The QIC expert team provided a set of pragmatic severity criteria, derived from DSM-IV and diagnostic instruments, to the clinicians. One aspect of the severity criteria was the duration of depressive symptoms. Discussing depression identification from the different professional perspectives was new to the clinicians, and it took some time to create a shared understanding of the conceptualization of depression in daily practice:

'Our cultures are different and we are quite convinced of our own treatment approach. One can have a psychiatric view of depression and a psychological one. To discuss this with an open mind, it needs time, but that is what happened.' (Team 7)

Many clinicians were positive about the new criteria for differentiation between patient categories, which seemed to help them develop their diagnostic skills:

'I find it remarkable that I was not used to the new terminology of severe depression and non-severe depression...Especially the criterion of time as a factor impacting on severity was an eye-opener to me when I joined the QIC...and I think for others too. I find this a refinement of my diagnostics and my clinical approach. This is an important advantage.' (Team 3)

Some PCPs preferred to keep old diagnostic styles, because of fundamental disagreement with the medical model underlying the diagnosis of depression. They gave a different meaning to the concept of depression than the QIC stepped-care model, especially to the milder forms, and rather looked at underlying problems instead of focusing on symptoms. For example, if a person developed depression following the loss of a beloved one or because of a chronic illness, the PCPs did not label and treat the depressive symptoms as a depression. Even though the QIC experts advised to include this category of patients in the project and offer them a self-help or preventive intervention, the clinicians often did not follow these instructions:

'It was difficult to include people in the depression project, because I often thought: if I solve the problem that causes the depression, the depression will disappear. Therefore, I did not interpret the problems as a depression, but rather as...a mood that corresponds to what is happening to this person.' (Team 1)

According to these clinicians, labeling and treating the symptoms as a disease could have the negative effect of adopting too narrow of an approach to the patient's problems, offering medical solutions without considering the patient's story and contextual factors. Another reason for not diagnosing depression was a good functional state of the patient. Some clinicians expressed that they would not discuss depression or bring the topic up when consulting with patients who still had high functioning.

The team discussions about the nature of the depression, as a part of the QIC method, was a learning experience for the team members, and a facilitating factor for further refinement of diagnostic skills:

'This is exactly the gain of working together, to look at depression in all its aspects, because one does not become depressive just like that, there is a whole story behind it, and if one only looks at the symptoms and treat those then one can make mistakes' (Team 6).

Our data show that many factors influenced the implementation of the first stepped-care principle of patient differentiation. Some can be related to the NPT constructs of 'coherence,' the process of creating a shared understanding about who is depressed, who is not and the severity level of the depression. This understanding needs to emerge in conjunction with the construct of 'cognitive participation,' the process to get clinicians actively engage with the depression work [20]. Both constructs were driving the implementation of patient differentiation. Although the boundaries of depression and the severity criteria were handed to the clinicians by the QIC expert team, the multidisciplinary teams went through an intensive process of exchange about the different perspectives on depression--the 'psychiatric' and the 'psychological' perspective. This process was time consuming, but finally resulted in the 'buy-in' of many clinicians into the stepped-care principle of patient differentiation, except for some PCPs who had difficulties applying the depression criteria to patients with mild, context-related symptoms (see Table 3 for an overview of the NPT related factors).

**Table 3:** QIC factors influencing the achievement of the NPT constructs and depression propositions

NPT constructs (May and Finch, 2009)	Corresponding propositions (Gunn *et al.*, 2010)	QIC factors
Coherence	Depression work requires conceptualization of bounderies (who is depressed, who is not depressed). Depression work requires techniques for dealing with diffuseness.	Facilitators:
		• The QIC stimulated multidisciplinary team discussions with open exchange of perspectives. The stepped-care model offered clinicians a technique for shared understanding on depression (who is severely and non severely depressed).
		• The BDI offered a framework for dealing with diffuseness of depressive symptoms.
		Barriers:
		• Different professional views on depression causing long discussions.
		• Disagreement of some clinicians with the medical model underlying the stepped-care model.

Cognitive participation	Depression work requires engagement with a shared set of techniques that deal with depression as a health problem.	Facilitators:
		• The new low intensity stepped-care treatment options fitted well into the primary care perspective.
		• The QIC meeting helped the exchange of the different views and come to agreements about the local depression care pathway and the task division.
		• Working with the stepped-care model improved the knowledge, skills and self confidence of primary care clinicians.
		• Treatment choices could be easily shared with the patients, leading to better working relationships.
		Barriers:
		• Unfamiliarity within the teams with each others skills and perspectives.
		• The negative attitude of some clinicians towards standardization of depression care.
		• The belief that (pro-active) monitoring is not a normal part of the PCP's work, and rather the patient's own responsibility.

Collective action	Depression work requires agreement about how care is organized, who is required to deliver care, and their structural and human interactions.	Facilitators:
		• The possibility to tailor the stepped-care model to the local setting.
		• Training was offered to apply the stepped-care interventions.
		• Regular team meetings to discuss individual treatment plans, helped to agree on how stepped care was delivered.
		• Competition between the different disciplines was not conceived as a problem because of the large amount of work to be divided.
		• Government policies have stimulated 'the stepped-care movement' over the last decade.
		Barriers:
		• Poor organizational infrastructures, such as the absence of links with specialty care.
		• A lack of funding of the new low intensive interventions, such as physical exercise.
		• A lack of patients opting for specific interventions.

Reflexive monitoring	Depression work requires the ongoing assessment of how depression care is done.	Facilitators:
		• Improved motivation because outcome measurement can structure and advance care for individual patients.
		• Positive reactions of patients and improved relationships, as a result of sharing the monitoring results.
		• Improved self-confidence of clinicians in making treatment decisions based on objective measurement.
		Barriers:
		• Multiple logistical problems for getting the questionnaires handed out and returned by the patients.
		• The absence of supportive systems (ICT, reminder systems) or staff.
		• The absence within the primary care teams of a culture and skills for process evaluation.

### Stepped treatment of depression

Most of the change activities of the primary care teams were spent on the second principle displayed in Table 1, stepped depression treatment. The implementation efforts were mainly directed at adding one or more low intensity treatment interventions to the usual routine practice, to serve as alternative treatment option to antidepressant medication for patients with non-severe depression. The stepped-care model implied that, alongside the introduction of new interventions, the teams standardized and structured their care for the different levels of depression (non-severe and severe). To some, this idea of structuring and coming to multidisciplinary agreements was helpful, because it gave them a sense of control over the care process. Others were hesitant about it, because they believed that depression care is difficult to standardize, because all patients have a unique symptom profile.

It took some teams quite a long time to discuss the stepped-care interventions, compare them to existing routines, study the 'toolkit' with descriptions of the interventions provided by the expert team, define the skills and capacity necessary to provide them, and reach agreement upon who should provide the interventions and how to implement them locally. This process was especially relevant in the multidisciplinary teams in which the clinicians were unfamiliar with each other's skills and work. Getting to know other team members was a first but necessary step:

'It is important that we now know what everybody has to offer...that process happened in harmony...so now I can refer even more adequately to social work, for instance, if I want the patient to have intensive coaching or practical help...that kind of care.' (Team 4)

The actual implementation activities consisted of different kinds of preparations for stepped depression care. Most teams installed a regular multidisciplinary staff meeting for discussions about patient care plans. Manuals, procedures, and patient information leaflets were developed, educational workshops were attended, new healthcare providers were contacted to provide specific care modules, and insurance companies were approached for additional funding. In addition, many team members participated in workshops offered by the QIC to train themselves in specific techniques, such as problem-solving treatment and psycho-education.

Despite the hard work, it was not possible for every team to organize one (or more) of the new interventions within the 15-month time frame of the QIC. However, if they did succeed to introduce new interventions, this allowed them to offer new choices to patients, as alternative interventions to medication:

'Well, I liked noticing that I did change my routines...When handling depression, I used to think: either I prescribe medication and do some consultations or I refer to specialty care. Now, many other options appear to be possible' (Team 7).

Changing routines in clinical practice for depression treatment had several benefits. Not only were team members able to offer more alternative interventions to clients, but they developed the self-confidence to do so, rather than simply prescribing antidepressants. The team members also felt that overall, changing routines for depression care was a positive experience:

'Many people have had good experiences with antidepressants. They find it hard to leave them. Also the PCP is used to prescribing them...but in the course of the project I progressively managed to offer alternative, low intensity interventions, because I started to believe in them myself...Nowadays I tell my patients that I keep antidepressants up my sleeve' (Team 8).

New interventions were not always provided according to the descriptions in the QIC toolkit. Some clinicians openly admitted to offer their patients depression care with 'a bit of everything.'

There were several PCP's who displayed mistrust in the effectiveness of the low-intensity treatment options. The reason for disagreement and mistrust stemmed from the fact that these PCP's believed that offering only low-intensity interventions implied insufficient treatment. This group of PCP's argued that patients with depression are in need of more substantial treatment options, and that 'just giving a self-help manual and tell them to return in six weeks, is not general medicine.'

As a consequence of the teamwork, the contacts between the clinicians within the improvement teams improved in terms of knowing and understanding each other, and facilitating more open and direct communication and a shared language. Clinicians experienced that this improved communication positively impacted on day-to-day collaboration and thought this change of itself had been one of the most important gains of the QIC:

'I think our collaboration improved...getting to know each other by spending time together. To me, improved collaboration, independent of depression care, has been an enormous gain of this project.' (Team 1)

According to the respondents, competitions between mental health nurses, social workers, and psychologists did lead to discussions, but did not result in real conflicts. The argument was that due to the vast number of depression interventions to be implemented, there was work to be done for all types of healthcare workers within primary care. Considering this, team members mostly preferred to be complementary instead of competitive. In relation to specialty care, complementary action included reaching an agreement with staff working at the specialist level, to refer patients to existing self help programs when necessary. Competitive actions included instances where several primary care teams established a new physical exercise group within primary care, rather than referring patients to existing programs at the specialist level in psychiatric facilities. This reasoning for the introduction of such a program was due to the belief among these primary care teams that bringing exercise 'to the patient' was a better response to address the needs of the patient.

Different factors influenced the implementation of the new interventions. Barriers for introducing them were poor organizational infrastructures in primary care, a lack of financing of some psychological or physical interventions, a shortage of patients with depression choosing the new interventions, and a lack of collaboration from specialist care organizations who were not always keen on sharing care. Other factors were facilitating the implementation of stepped care, for example national policies and regulations within the healthcare system. Some respondents spoke about 'the stepped-care movement' that started about ten years ago, but only recently came to reality due to multiple favorable conditions coming together. The QIC had given this movement a 'push,' and although the implementation of the full stepped-care model did not occur within the given time frame, change in the right direction did occur in the eyes of the teams:

'It is very difficult to induce change in a short period of time, I have noticed. On the other hand, I did sense enthusiasm for this very workable model...It mainly 'structures' the care that a PCP provides and creates possibilities for agreements. Yes, I do feel positive about this, it would be a waste to return to old routines again, and that's what I notice amongst my colleagues as well.' (Team 4)

These results show that the second principle of the stepped-care model--implementing stepped-care treatment--was mainly translated by the clinicians in trying to introduce new interventions and reduce antidepressant prescribing. This process demanded an intensive process of 'cognitive participation' and 'collective action,' engagement with a shared set of techniques and agreement on how the work should be organized. Collective action, according to Gunn *et al. *is defined as 'purposive action aimed at a clear goal, and is influenced by both organizational (external) factors and immediate (internal) factors [20].' Important positive internal factors in our data were related to the clinicians developing trust and good relationships among the team members and with patients, important external factors were related to poor reimbursement of the new interventions, and stimulating stepped-care policies helping the implementation process. (Table 3).

### Outcome monitoring of depression

The third stepped-care principle to be implemented consisted of structural use of the Beck Depression Inventory (BDI), which had to be completed every six weeks by the patient until a score of 10 or lower was achieved [25]. This implies that in case of a no-show of a patient, the team needs to pro-actively contact and follow-up with the patient. The structural use of a depression measurement within the QIC context served two functions. First, it served as an outcome indicator within the stepped-care model, to follow-up on the patient's well-being, and step up to a next treatment level if the patient was unresponsive. This use of the BDI was much appreciated by the clinicians:

'Such a measurement instrument in a primary care practice is very special. And in fact, I do feel positive and enthusiastic about it. To follow the course of the depression in this way and the treatment time..three months, six months...and if something does not work, one can take the following step.' (Team 4)

The second function of the BDI was to serve as a process indicator, in an effort to help the improvement team reflect on the progress of the implementation process, to identify barriers and adapt implementation strategies where necessary. This introduction of the use of the BDI instrument as a process indicator was part of the PDSA cycle, a formal component of the QIC method. The teams were asked to use process indicator patterns and trends over time to reflect on their implementation work, but this method of making goals and processes explicit and accountable did not appear to fit within the more intuitive cultures of the primary care teams:

'I did not need the Plan-Do-Study-Act method, neither did my group. Rather, it created confusion...What we did, we just started and tried to profit off each other's added values...and of course we tried to improve the care for patients with depression. We simply worked with that in mind and that was all we needed.' (Team 2)

There were a number of barriers to the introduction of the BDI, as the structured use of a patient questionnaire for depression was virtually unprecedented. Baseline BDI measurement, at the beginning of care, was relatively easy to organize compared to repeated measurement. One professional-related barrier discussed during team meetings was the vision of some teams that depression measurement on a continuous basis by pro-actively asking patients to fill out the BDI, was 'patronizing' and therefore not in accordance with a PCPs professional role but more appropriate for other roles, like social workers. Arguing that monitoring is the patient's own responsibility was cited as another reason for not ensuring that the BDI was continuously registered over time:

'Sometimes I see someone with a BDI of 20, and in spite of this I still conclude that this is not depression...Some weeks later the person visits me again and I see that things have calmed down. And after that the person does not turn up again...In those cases I do not call the patient myself, that is not my way of working. I consider that to be somebody's own responsibility' (Team 3).

Although the clinicians clearly invested time and effort to use the BDI as a monitoring tool and attempted to make it work, organizational barriers made the use of the BDI a very time consuming and difficult task. Having the BDI sent to the patients and returned to the practice, the lack of supportive Information and Communication Technology (ICT) for reminding the clinicians about the BDI or for registration and feedback of BDI scores, and a lack of administrative staff, were hindrances to BDI implementation. Despite these difficulties, some clinicians did manage to incorporate the instrument into their work processes. The patients' reactions to this were surprisingly positive, despite the prior expectations of many that patients would not co-operate. While using the instrument during consultations, the patient-doctor communication became more structured, focused, and therefore more meaningful for both. This was an unexpected function of the BDI instrument:

'I found patients to be very enthusiastic about the BDI. You wonder how they will react when you give them a questionnaire like that. Well, very positively. And for a PCP it provides a starting point for the next consultation, something to talk about, a lead....'(Team 4)

Additionally, another also unexpected function of the instrument was the clinicians' perception that the BDI legitimated treatment decisions and gave some objectivity to them. Like a thermometer indicating the patient's fever, the BDI made the clinicians feel more certain in decision-making, confirming that they were not 'just doing whatever came up.' At times, the clinicians noticed that this 'objectivity' also worked out positively for patients as well, particularly when their BDI score changed to a lower score. A declining score served as a hopeful message to the patient, as 'proof' that the depressive symptoms were going to go away even where the patient had not yet experienced any symptom improvement. However, clinicians did not always trust the BDI score and sometimes valued their subjective assessment of the client as more important, thus relying on their own clinical judgment.

Relating back to the NPT constructs, the implementation of the BDI during the QIC does not correspond to the construct of 'reflexive monitoring,' which is the notion that depression work demands an 'ongoing assessment of how depression care is done' [20]. Reflexive monitoring, in terms of using data for understanding the implementation process and guide discussions that may lead to modification of the implementation goals and strategies, did not occur as intended by the expert team, mostly because it did not match with the primary care culture for introducing change. While teams did introduce the BDI, the function was more to appraise well-being and the treatment plans of the patients, rather than using it as a tool to measure progress and process. Different factors, related to other NPT constructs, influenced the actual implementation of the BDI (Table 3).

## Discussion

The introduction of a stepped-care model for depression to primary care teams within the context of a depression QIC was generally well received by the participating clinicians. All three elements of the proposed stepped-care model (patient differentiation, stepped-care treatment, and outcome monitoring) were translated and introduced locally. The process was influenced by a complex set of factors. Facilitating factors for the implementation process was the stepped-care model itself, the structured team meetings as part of the QIC method, and the positive reaction received from patients to stepped care. Hindrances to rapid implementation included the differing views of depression and depression care within the multidisciplinary healthcare team, lack of resources, and underdeveloped information systems. As a result of these hindrances, clinicians were not able to fully adopt the stepped-care model as a new treatment approach embedded in primary care, but did manage to take some strides towards utilizing this treatment approach. The stepped-care changes reported by the clinicians were: learning how to differentiate between patient groups with depressed symptoms and different levels of care; being able to offer a broader treatment package to depressed patients including low intensity interventions; changed antidepressant prescribing routines; and better working relationships with patients and with colleagues.

Although all four NPT constructs operated concurrently in the QIC, 'coherence' and 'cognitive participation' appeared to be crucial drivers, especially in the beginning of the process. The introduction of the stepped-care model by the expert team was not enough to get the clinicians started. The teams needed time for discussions and information exchange to reach a shared understanding of depression and depression care and to come to local agreements about the selection of interventions and the distribution of tasks amongst the different team members. In teams where members did not know each other prior to the QIC, it was a very time-consuming process to reach a shared understanding of depression care and get clinicians engage with the change process. The stepped-care model itself provided clear guidance for 'collective action' and the actual implementation of new interventions for depression, but external factors such as poor financing hindered the change process. The NPT construct of 'reflexive monitoring' did not happen as explicitly as the QIC method intended. Instead of following PDSA cycles, supported by monitoring results, the teams moved on rather intuitively, using the BDI data to follow patients outcomes and adapt the treatment plan accordingly.

### Relation to other studies

In our study, we found that a shared understanding of depression and depression care is a crucial step towards change. This is in line with the view of Gunn *et al.*, who argue that primary care clinicians 'hold a different view of depression and depression work compared to the traditionally applied psychiatric viewpoint' and suggest that 'without shared agreement about what primary care means by the term depression, diagnosing and developing adequate treatment and management pathways will remain difficult' [20].

The QIC intervention relates to other national depression quality improvement work in primary care, such as the research by Meredith *et al.*, a process evaluation of a American depression QIC, based on Wagners chronic care model, with multidisciplinary quality improvement teams in 17 diverse primary care organizations [26]. The evaluation comprised semi-structured interviews, conducted with team leaders, about the successes and the barriers that facilitators experienced during the QIC. Results revealed that some elements of the chronic care model changes were adopted by all the teams (proactive follow-up, patient education, patient registry systems, and care planning), while other changes were not (provider participation and patient activation). The only barrier that affected perceived success was poor leadership support.

Another similar initiative is the RESPECT project, which involved depression care management, collaboration between mental health and primary care professionals, and preparation of primary care clinicians and practices to provide systematic depression management. Patient response was monitored with the Patient Health Questionnaire-9 (PHQ-9) [27]. Results from the interviews revealed that a lack of reimbursement posed the greatest obstacle for implementation of the care model. Successful dissemination of the depression care model was found to be related to a broadly shared vision and commitment at all levels of an organization, clearly articulated by clinical leadership and a systematic change strategy in place to improve chronic care [28].

Our results are also in line with a recently published report of a stepped-care implementation study in the United Kingdom, concluding that implementation of stepped care at different sites varies greatly according to different contexts [24]. Richards *et al. *suggest that 'prescriptive national initiatives should incorporate local modeling to translate national prescriptions to specific situations' [29]. Although the implementation intervention applied in this project did not contain local computerized modeling, it did incorporate a modeling process in terms of creating time and support for primary care teams to discuss and translate the stepped-care principles to their own contexts.

### Strengths and limitations of the study

Strengths of this study are the depth and detail generated about the introduction of stepped care amongst a fairly large and diverse group of primary care clinicians, who actually experienced these change processes. The use of NPT helped to interpret the findings in a generalizable framework for adoption of new routines. A limitation of our work is that our findings are based on experiences of clinicians who volunteered to participate in a quality improvement program and were supported to implement a stepped-care approach. Our findings might be less applicable outside the context of a quality improvement project. Secondly, we only interviewed the improvement teams once during the course of the program. Additional interviews at different time points could have reduced the risk of missing information that would have been important to understanding the implementation process; however, we believed one interview was sufficient to capture experience with the implementation process. Furthermore, financial and pragmatic constraints did not allow for multiple interviews.

### Interpretations of the findings and implications for practice

Our data strengthen our expectations that the introduction of stepped depression care within primary care teams is time consuming, and dependent on an interaction of complex factors. We found barriers and facilitators for this change at different levels: the stepped-care model and the QIC itself, the individual professional, the patient, the social context, the organizational context, and the economic and political context. Despite these barriers, the QIC context helped the teams to move towards a more stepped-care approach for depression, using the three principles of the QIC model. Our data underscore the unpublished quantitative findings of the QIC intervention study--that it seems unlikely that the changes reported by the clinicians would have occurred by itself within the primary care teams. The data show that the processes of coherence and cognitive participation within the multidisciplinary teams were so crucial, and that without the QIC infrastructure of regular and structured multidisciplinary team meetings, coordinated by a local project manager, the stepped-care model might not have been emphasized so much and translated to local circumstances by the clinicians as it was during the QIC.

This raises the question whether providing a care model and organizing team meetings are sufficient to induce change or whether clinicians also need supportive mechanisms such as following PDSA cycles or making use of an expert team. According to our data, the explicit use of PDSA cycles and the expert team seemed to be of less value to the primary care teams. Another question that remains is whether QICs in mental health, instead of taking a rather one-size-fits-all approach, should be designed in a more flexible way and show more sensitivity to local problems in terms of poor 'coherence' or 'engagement' of individuals. Additional implementation studies may be able to answer these questions and make suggestions for adaptations of the QIC method.

The take-home message, based on our additional NPT analysis, is that future implementation projects for depression in primary care should incorporate sensitizing strategies for addressing local problems of participating teams. Facilitators who guide these processes should be particularly attentive to local problems related to 'coherence' and ' cognitive participation.' If implementation strategies are too oriented towards action and rapid implementation, clinicians might not engage. Also, systems for reflexive monitoring are still not implemented and need to be addressed if policy makers aim at processes of continuous quality improvement in depression care [20,30]. Besides this message that the use of NPT has led us to, the application of the NPT constructs to our data has also been problematic due to the overlap and difficulty of discerning the difference between the constructs. Another criticism of NPT is related to the point that May and Finch address: that NPT 'focuses on the work of embedding and of sustaining practices within interaction chains' [19]. This implies that the NPT constructs are mainly based on perceptions of people, which presents the risk of leaving some contextual factors beyond the scope.

Stepped depression care has been strongly promoted throughout the Netherlands in the past five years. Both the stepped-care model used in our study, as well as several of the QIC method elements have been helpful tools to guide stepped-care implementation in some regions. It is important to consider that users of the model might consider an adaptation such as the adoption of the PHQ-9 instead of the BDI. The BDI was selected by the QIC expert team in 2004, based on their own experience with this instrument in specialist care. However, the PHQ-9 has become more widely used by now, and has some advantages over the BDI, such as its ability to diagnose and monitor the severity of depression, in addition to its brevity and usefulness in primary care practice [31]. An adaptation of the QIC method may be to use the NPT constructs to check progress of change, to identify team barriers, and to develop strategies to overcome these barriers. These adaptations of both the stepped-care model as well as the QIC method could be topics for future research in this area.

## Conclusions

Stepped depression care can be received positively in primary care. Although implementation of a full stepped-care approach cannot be reached within a short time frame, within a QIC context clinicians can set steps into this direction. Creating a shared understanding within multidisciplinary teams of what constitutes depression and coming to an agreement on the content of depression care and the division of tasks is important to address during stepped-care implementation processes.

## Competing interests

All authors have completed the Unified Competing Interest form at http://www.icmje.org/coi_disclosure.pdf (available on request from the corresponding author) and declare: no support from any organisation for the submitted work; no financial relationships with any organisations that might have an interest in the submitted work in the previous three years, no other relationships or activities that could appear to have influenced the submitted work.

## Authors' contributions

The research was designed and initiated in 2005 by GF, MW, and RG. Prior to that GF was involved in the process of designing the stepped-care model with a depression expert panel. The model was piloted by GF during a first depression QIC in different regions prior to the QIC researched in our study. The study interviews were all conducted by GF and or MO, without them being involved in the QIC. GF, MO and JdL were involved in data-coding, whereas JdL also was the project-leader of the national QIC expert team. All authors had full access to all of the data (including tables) in the study and can take responsibility for the interpretation and publication of data used within this paper. All authors were fully independent from the funders.
